# Donor Age, Sex, and Cause of Death and Their Relationship to Heart Transplant Recipient Cardiac Death

**DOI:** 10.3390/jcm12247629

**Published:** 2023-12-12

**Authors:** Margo E. Hammond, Charles Zollinger, Andrija Vidic, Gregory L. Snow, Joseph Stehlik, Rami A. Alharethi, Abdallah G. Kfoury, Stavros Drakos, M Elizabeth H. Hammond

**Affiliations:** 1 Department of Biochemistry, Brigham Young University, Provo, UT 84602, USA; mbethhammond@gmail.com; 2 Intermountain Donor Services, 6065 S Fashion Blvd, Murray, UT 84107, USA; chuck.zollinger@donorconnect.life; 3 Department of Cardiology, University of Kansas Hospital, 4000 Cambridge St., Kansas City, KS 66160, USA; andrijavidic2010@gmail.com; 4 Department of Statistics, Brigham Young University, Provo, UT 84602, USA; greg.snow@imail.org; 5 Department of Cardiology, University of Utah Hospital, 50 N Medical Drive, Salt Lake City, UT 84132, USA; josef.stehlik@hsc.utah.edu (J.S.); stavros.drakos@hsc.utah.edu (S.D.); 6 Cardiac Transplant Program, Intermountain Medical Center, 5252 S Intermountain Drive, Salt Lake City, UT 84157, USA; rami.alharethi@imail.org (R.A.A.); boudi.kfoury@imail.org (A.G.K.)

**Keywords:** innate immunity, intracranial hemorrhage, heart transplantation, donor factors, antibody mediated rejection, cardiovascular death, allorecognition

## Abstract

Background: Recent studies indicate that donor innate immune responses participate in initiating and accelerating innate responses and allorecognition in the recipient. These immune responses negatively affect recipient outcomes and predispose recipients to cardiovascular death (CV death). We hypothesized that a donor cause of death (COD) associated with higher levels of innate immune response would predispose recipients to more adverse outcomes post-transplant, including CV death. Methods: We performed a single-institution retrospective analysis comparing donor characteristics and COD to recipient adverse cardiovascular outcomes. We analyzed the medical records of local adult donors (age 18–64) in a database of donors where adequate data was available. Donor age was available on 706 donors; donor sex was available on 730 donors. We linked donor characteristics (age and sex) and COD to recipient CV death. The data were analyzed using logistic regression, the log-rank test of differences, and Tukey contrast. Results: Donor age, female sex, and COD of intracranial hemorrhage were significantly associated with a higher incidence of recipient CV death. Conclusions: In this single institution study, we found that recipients with hearts from donors over 40 years, donors who were female, or donors who died with a COD of intracranial hemorrhage had a higher frequency of CV death. Donor monitoring and potential treatment of innate immune activation may decrease subsequent recipient innate responses and allorecognition stimulated by donor-derived inflammatory signaling, which leads to adverse outcomes.

## 1. Introduction

Cardiac transplantation is an effective therapy for cardiac failure in many patients [1]. Recent improvements in immunosuppressive treatments have decreased acute cardiovascular (CV)-related mortality in transplant patients [2]. However, cardiac allograft vasculopathy (CAV) resulting in CV-related death (CV death) continues to be a serious cause of allograft loss in patients surviving the initial posttransplant period [2,3]. Recent studies indicate that both donor and recipient innate immune responses (IR) participate in initiating and accelerating recipient allorecognition (AR), which can lead to CAV and CV death [3,4,5,6,7,8,9,10]. Thus, it is likely that both donor and recipient IR are important participants in the pathogenesis of CAV and CV death [5,6,7,8,9,10]. We hypothesized that donor characteristics and causes of death (COD) with higher IR would have an adverse effect on recipient outcomes, including the incidence of CAV and CV death. We performed a single-institution retrospective analysis comparing specific donor characteristics associated with heightened donor IR to recipient outcomes. We chose to compare donor factors of significance based on previous publications, including donor age, sex, height, weight, COD, and ischemic time. We used CV death as an outcome variable for the study [11,12,13,14,15].

## 2. Methods

### 2.1. Database

We obtained institutional review board (IRB) permission to review the donor records of all available cardiac transplant recipients from 1987–2016 in the Intermountain Donor Services (IDS) files. Donors included in the study were those whose records included sufficient information about donor details to allow analysis. The records included demographic characteristics, COD as recorded by the donor institution, results of antibody screening, infectious disease evaluation, history of autoimmune disease, alcohol use, smoking history, and drug use if available. The information was uploaded to a FileMaker Pro 12 (FileMaker, Inc. Santa Clara, CA, USA) database created for this purpose. The Utah Transplant Affiliated Hospitals (UTAH) cardiac transplant database for recipients includes demographic characteristics and details of recipient pathology and outcomes since 1987 [15]. Recipient follow-up intervals ranged from 6 to 35 years (up to 2022).

### 2.2. Patient Population

We analyzed anonymized records of all local adult (age 18–64) donors in the IDS database with transplant dates between 1987 and 2016. Donor records without sufficient clinical information were excluded. We clustered the differing CODs into three categories. The first category contained CODs with traumatic brain death (TD) involving a motor vehicle (MVA), localized traumatic head injuries such as blunt trauma (BHT), or a gunshot wound (GSW) to the head. The second category of COD was anoxic brain injury, including drowning and drug-related deaths. The final category was intracranial hemorrhage (ICH). We linked these records with their corresponding recipients and recipient outcomes in our database.

### 2.3. Statistical Analysis

We compared donor age, COD, and sex with the frequency of recipient CV death or retransplantation (according to UNOS criteria) using logistic regression, a log-rank test of differences, and Tukey contrast. R version 4.3 (2023) was used for the analysis. *p*-values < 0.05 were considered significant. UNOS criteria for CV death include death from acute rejection, acute myocardial infarction, acute arrhythmia, heart failure, or CAV [14,15]. For the analysis of the relationship of various CODs to CV death, the CODs were grouped as anoxia, traumatic death (TD, which includes MVD, BHT, and GSW), and ICH. The log-rank test tests whether the 3 groups have similar survival patterns and then computes the expected number of CV deaths in each group based on the time that the patients spend in the cohort (time to CV death or censoring). That is the expected category. Then, it compares the actual number of CV deaths to the expected number for each group [16].

## 3. Results

### 3.1. Donor and Recipient Characteristics

Donor age information was available for 706 of the 1294 adult recipients in the data set, and donor sex information was available for 730. The mean donor age was 29.2 ± 16.9 years. The mean recipient age was 49.9 ± 18.1 years. A total of 136 of the donors were over 40 years old. In addition, 692 (55.7%) of the recipients died during the study interval. The average time from donor admission to the hospital to transplant was 58.2 h. Two donors had a history of cancer, 7 had a history of hypertension, 15 had some form of autoimmune disease, 5 were diabetic, 93 smoked, 115 drank moderately or heavily, and 80 had a history of drug abuse. None of the donors had active infections at the time of transplantation. No information was available about preformed antibodies. Information about immunization responses was also unavailable. All donors included were local, so the ischemic time was under 4 h for each transplant. Recipients and local donors were matched by height and weight within 25% of each measure. 

### 3.2. Effect of Donor Age on Recipient CV Death

**Figure 1:**
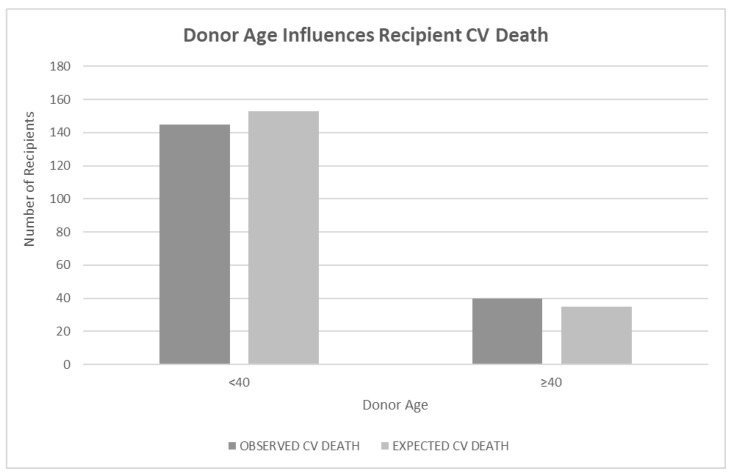
Legend: Bar graph showing the relationship of donor age to recipient cardiovascular (CV) death outcomes. Donor age was defined as <40 or ≥40 years at the time of transplant. Bars represent the observed CV death rate next to the expected CV death rate. Information about donor age was available for 706 donors. Age ≥ 40 was statistically significant based on a log-rank test of differences (*p* < 0.05).

When donor age was investigated as a continuous variable using a Cox proportional hazard regression with the CV death of the recipient as the outcome and donor age as the predictor, the result was insignificant (*p* < 0.24). A log-rank test of differences using donor age < 40 years old and ≥40 years old as a predictor of recipient CV death showed a significant difference in CV death of recipients if the donor was older than 40 years old (*p* < 0.05) (Figure 1).

### 3.3. Effect of Donor Sex on Recipient CV Death

**Figure 2:**
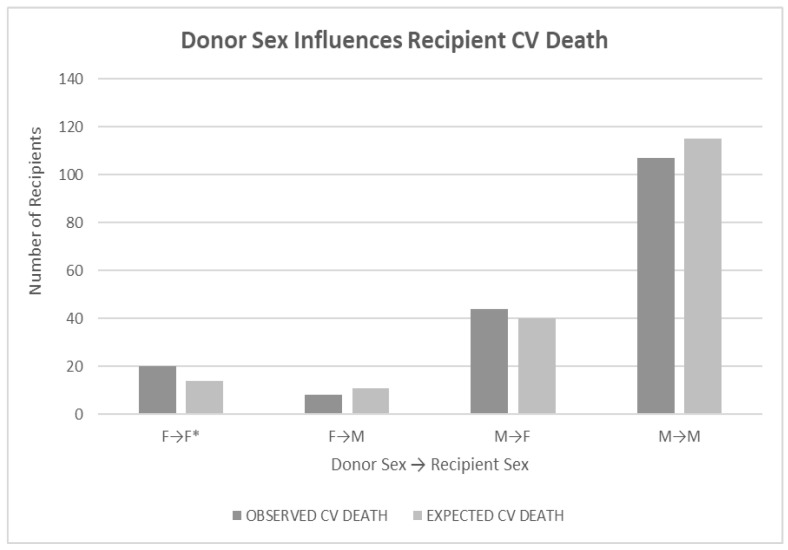
Legend: Bar graph showing the relationship of donor sex to recipient CV death outcomes. Recipients with female donor hearts died more frequently from CV causes (*p* < 0.05). The starred relationship highlights the significant comparison. Recipient sex was not as influential (*p* < 0.09). Analysis was done using a log-rank test of differences. Evaluation of donor-recipient sex mismatches also showed no significance for any combination (*p* < 0.62). * highlights the significantly worse mortality of female donor hearts transplanted into female recipients.

Recipients with female donor hearts experienced CV death more frequently (*p* < 0.05) if the recipient was female (*p* < 0.05), but the result was not significant if the recipient was male (the starred comparison in Figure 2). Recipient sex was not as influential on CV death (*p* < 0.09). Analysis was performed using a log-rank test of differences. Evaluation of donor-recipient sex mismatches also showed no significance for any combination (*p* < 0.62).

### 3.4. Effect of Donor COD on Recipient CV Death

**Figure 3:**
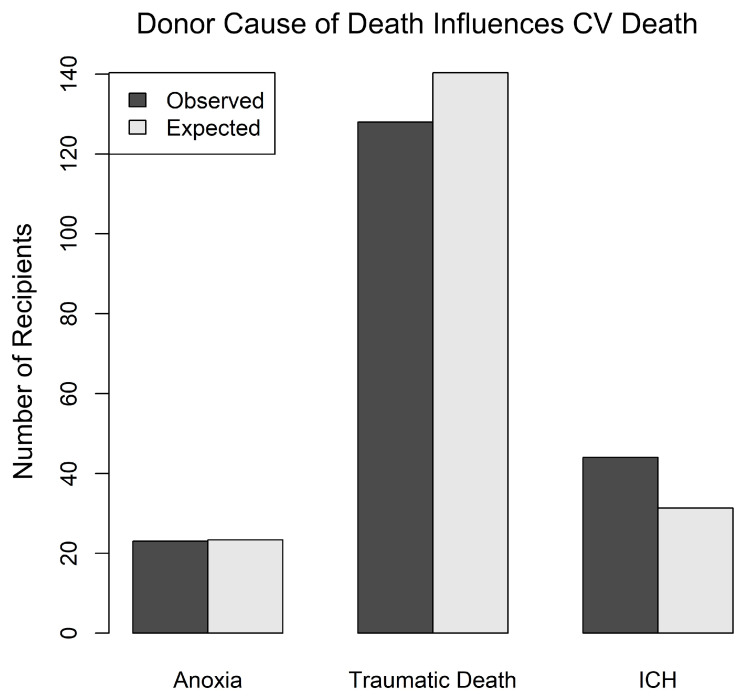
Legend: Bar graph showing the relationship between donor cause of death (COD) and recipient CV death outcomes. The analysis was performed using a log-rank test of differences, where COD was the predictor of death. For this analysis, CODs were grouped as anoxia, traumatic death TD (including GSW, MVA, and BHT), and ICH. The log-rank test tests whether the three groups have similar survival patterns and then computes the expected number of CV deaths in each group based on the time that the patients spend in the cohort (time to CV death or censoring). Patients with ICH were significantly more likely to die of CV death than those in other categories: *p* ≤ 0.04. Anoxia = anoxic death; GSW = gunshot wound to the head; MVA = motor vehicle accident, including any vehicle and auto-pedestrian accidents; ICH = intracranial hemorrhage; BHT = other causes of blunt head trauma; TD = traumatic brain death.

A donor COD of ICH was associated with an increased incidence of CV death in the recipient. The analysis was performed using a log-rank test of differences, where COD was the predictor of death. CODs were grouped as anoxia, traumatic death TD (including GSW, MVA, and BHT), and ICH. The log-rank test tests whether the three groups have similar survival patterns and then computes the expected number of CV deaths in each group based on the time that the patients spend in the cohort (time to CV death or censoring). Patients with ICH were significantly more likely to die of CV death than those in other categories (*p* ≤ 0.04) (Figure 3).

## 4. Discussion

In this study, we confirmed the adverse influence of older donor age and female sex on CV deaths among heart transplant recipients. Female donor hearts were more frequently associated with worse recipient outcomes in both this study and other published reports [15,17,18,19]. We have previously reported that female recipients were more likely to die after transplant from antibody-mediated rejection (AMR) or CV death [15]. Females have a higher immune response based on both genetic and hormonal influences [20,21]. Numerous cell types bear estrogen receptors on their plasma membranes, including endothelial cells, neuronal cells, hematopoietic stem cells, and mature immune cells [22,23]. Estradiol can bind to these cell types and trigger hormone-responsive genes and cytokine release, which directly affects the strength of both the innate and adaptive immune responses. Estradiol promotes the production of interferon alpha, regulates the function of innate immune cells (especially B cells, dendritic cells, and macrophages), and alters dendritic and macrophage maturation and activation. In addition, estradiol activates the coagulation system [24,25]. Therefore, female donors are expected to have a stronger immune response and trigger a correspondingly strong immunological response in recipients. By contrast, the actions of testosterone are largely anti-inflammatory and immunosuppressive [25].

A recent study of donor and recipient age found that older donor age was associated with higher recipient mortality, particularly in the short term [26]. We found a significant association between CV death and donors of either sex who were 40 years old or older. Emerging evidence suggests that during aging, chronic sterile low-grade inflammation (so-called inflammaging) develops and contributes to the pathogenesis of age-related diseases, including atherosclerosis, Alzheimer’s disease, type 2 diabetes, and cancer. Thus, the interaction we see between ICH and increased CV death may be related to the enhanced inflammasome activation triggered by hypoxia and resultant ROS activation through inflammaging in older recipients [27,28].

In this limited study, only donor COD of ICH predisposed recipients to CV death. Traumatic death of other types (MVA, BHW, and GS) did not have a similar effect on CV death, which was unexpected. Anoxic brain death was also not associated with increased CV death.

ICH produces a brisk inflammatory response. The results of this study suggest that the inflammatory response is higher than that of traumatic death, another potent cause of innate immune signaling. ICH results in localized hypoxia and injury. During reperfusion of ischemic tissue after ICH, marked innate immune responses are triggered. This inflammation and its resulting cytokines activate microglial cells, induce the recruitment of bloodborne immune cells into the area of infarction, and upregulate inflammatory responses in neighboring endothelial cells. This triggers neutrophil accumulation and further activation of the complement and coagulation systems [29,30]. 

The activation of innate immune mediators in response to cellular stressors, including hypoxia and reperfusion, as seen in ICH, has been termed inflammasome triggering [28,29,30]. Components of inflammasome triggering include danger-associated molecular patterns (DAMPs) such as heat shock protein 70 (Hsp70), complement components, reactive oxygen species (ROS), signaling molecules such as NF-kB, pattern recognition receptors (PRR) such as toll-like receptors (TLRs), cytokines such as IL-6 and TNF, chemokine receptors, adhesion molecules, neutrophils, macrophages, and dendritic cells [18,19]. Many of these factors, notably NF-kB pathways, are also implicated in the pathogenesis of CAV and other forms of CV death after transplantation [3,4,5,6,7,8,9,10]. 

Similarly, donors who died from trauma (TD) had more tissue injury than donors dying from anoxia (usually drug overdose). These injuries have been reported to result in increased inflammatory responses, similar to those seen in donors dying of ICH [2,9,17,30]. Trauma causes diffuse blood pooling, resulting in extensive hypoxia, which is highly inflammatory. Additionally, recent studies in trauma patients have documented the existence of complement effectors, including C5a-C9, which peak 6 h after injury and remain elevated for several days [31,32,33].

**Figure 4:**
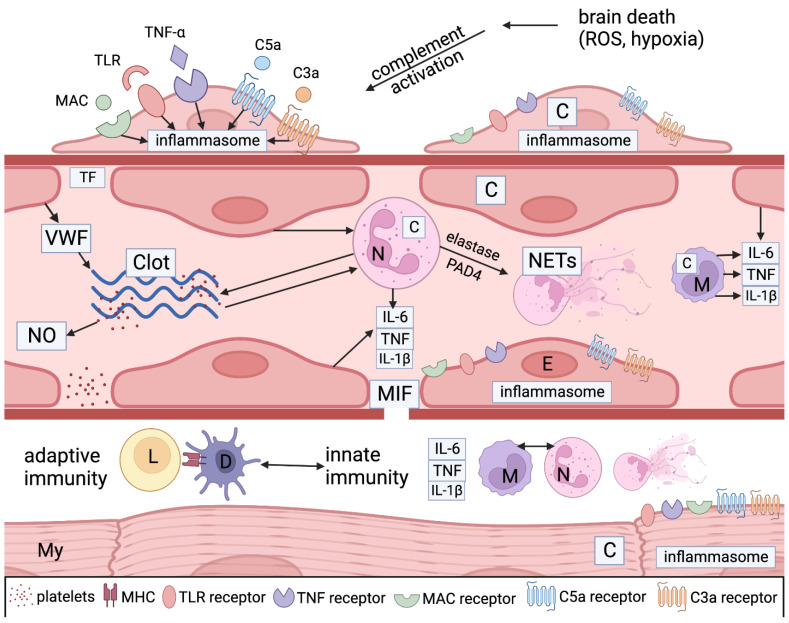
Legend: A simplified graphical illustration of the mechanisms of injury in donors after brain death. Ischemic stress and cell death lead to complement activation through the classical, alternative, and lectin pathways. DAMPS, such as the TLR ligand, TNF a, and complement components like C3a, C5a, and C5b-9 (MAC), bind with PRR receptors on pericytes (P), endothelial cells (E), neutrophils (N), and macrophages (M) to induce inflammasome assembly and release pro-inflammatory mediators like tumor necrosis factor alpha (TNFa), interleukin 6 (IL-6), and interleukin 1 beta (IL-1b). Microvascular endothelial cells, pericytes, myocytes (My), and inflammatory cells make more complement proteins (C) and inflammatory cytokines as well as macrophage inhibitory factor (MIF), which leads to gaps in the basement membrane, promoting vascular permeability and extravasation of neutrophils and macrophages and activating dendritic cells (D). Neutrophil activation and neutrophil extracellular trap (NETS) generation (through the action of elastase and PAD4) also play a pivotal role. They stimulate coagulation and complement activation, creating positive feedback loops between endothelial cells and platelets. Platelets generate nitrous oxide (NO), another potent mediator. Endothelial cells elaborate von Willebrand factor (VWF), which is an important accelerator of coagulation. Cell necrosis caused by the autoinflammatory process exposes cellular HLA antigens that initiate allorecognition by involving donor macrophages and dendritic cells (D), as well as potential autoimmunity against myocyte and endothelial antigens. Myocyte injury can be initiated by ischemia generated by the vascular injury and by adaptive and autoimmunity against myocyte donor-specific antigens (MHC) and others.

These mechanisms are highly relevant to recipient immune responses, such as AMR leading to CV death. Studies on myocardial ischemia and cardiomyopathy show that heart tissue is known to accelerate complement activity as myocytes and pericytes form complement components, which then accelerate injury from complement effectors and neutrophils recruited by this reaction [8,31,33] (Figure 4).

## 5. Continued Discussion

Recent studies highlight that complement activation occurs within 6 h of trauma or ICH and includes the triggering of classical, lectin, and alternative pathways of complement activation that are associated with neutrophil and platelet accumulation, as well as coagulation system activation [17,30]. This likely occurs with reperfusion as well. Complement receptors sense complement effector molecules (e.g., C3a, C5a, C3b, iC3b, and C3d) generated by DAMP-mediated activation of complement. Once activated, stress-induced signaling through receptors on endothelial cells, myocytes, and infiltrating leukocytes mediates continuous tissue injury. Complement receptors on antigen-presenting cells and T cells promote donor-specific allorecognition [19]. Effector responses against donor antigens are also enabled by complement and toll-like receptors (TLR), which accelerate the adaptive immune response. Complement effector molecules recruit more neutrophils, which generate further complement and coagulation responses [3,4,5,6,7,8,9,10,18,19]. Such responses are typically ongoing and unopposed without treatment until the time of transplantation. It is not surprising that these responses in donors can mediate further damage in recipients after transplantation.

## 6. Limitations

This was a single-center study, which limits statistical power. All donors and recipients were from the same region. Many donor records had missing information, which limited the number of patients that could be included in the analyses. Although we carefully reviewed each donor record, some information that may have influenced the donor outcomes was unavailable. Our analysis was also limited by the lack of information about donor hs-troponin levels, as well as the status of panel reactive antibodies and donor-specific antibodies. Other studies we have reported have previously evaluated other recipient factors [14,23]. Finally, our population size was too small to identify all potential interactions and evaluate them with sufficient statistical power.

## 7. Conclusions

In this single institution study with a long recipient follow-up of up to 35 years, we found that recipients with hearts from donors over 40 and female donors transplanted into female recipients had a higher frequency of CV-related deaths. These findings confirm previous studies. We also found that donors who died with a COD of ICH had a higher frequency of CV-related deaths. Age, female sex, and COD of TD or ICH in the donor have been reported to promote IR triggering and inflammasome activation as well as complement activation and effector activity, which are likely important drivers of CAV and CV death. While our results are suggestive, other prospective studies involving larger cohorts will be needed to expand our understanding of inflammasome activation and pathways and their potential uses in therapeutic strategies. We recognize that the cardiac transplant donor pool is limited. Our findings suggest that modified donor protocols to monitor markers of innate immune triggering like complement components and IL-6 in patients who are either female donors or those whose COD was ICH may predict which patients would benefit from emerging therapeutic strategies. Allorecognition by donor cells is an important accelerator of adaptive immunity as well. This donor function has recently been highlighted in a heterologous mouse transplant model where donor-derived macrophages provide most allorecognition [34].

More extensive donor evaluation and monitoring may suggest potential therapeutic interventions to improve recipient cardiac transplant outcomes.

## Data Availability

The data that were analyzed for this manuscript is housed in an Institutional Review Board (IRB)-approved REDCAP database at Intermountain Healthcare. A review of these files can be requested by Daniel Bride at daniel.bride@imail.org.
